# “It’s not all in my head!” - The complex relationship between rare diseases and mental health problems

**DOI:** 10.1186/s13023-017-0591-7

**Published:** 2017-02-27

**Authors:** Rebecca Nunn

**Affiliations:** University of Cambridge, Cambridge, UK

**Keywords:** Patient experience, Rare disease, Mental health

## Abstract

The incidence of mental health disorders is significantly higher in individuals with a rare disease, compared to the general population. This letter considers the possible reasons for this in terms of the many ways in which a rare disease impacts on an individual’s life, and how these impacts can be strongly related to factors which predispose to mental health difficulties.

Furthermore, issues surrounding mental health can also play a significant role in the process of diagnosing a rare disease. The unusual nature of such diseases intrinsically predisposes an individual to obtain an inaccurate diagnosis of a psychosomatic disorder, a diagnosis which can often be further complicated by the presence of genuine psychiatric symptoms.

This letter argues that these common experiences of rare disease patients have impacts upon the way in which their psychiatric care should be offered and managed, and that sensitivity and understanding surrounding these issues should be considered a necessary part of effective care for rare disease patients.

## Main text

As a person with a rare disease, one way in which I view my life is as a series of precarious balances. Balancing the need for a clear diagnosis with the need to get on with life. Balancing the time spent in hospitals as a patient with the time spent studying there as a medical student. Balancing the wish to have completely honest conversations with doctors with the worry that they will no longer take you seriously if you do so. Rare disease patients throughout the UK consistently report [1] that the effects of their condition stretch vastly beyond the medical, and have a large impact on many aspects of their lives. When these impacts are considered in the context of widely recognised risk factors for the development of mental illness [2], it starts to become clear why 69% of rare disease patients report experiencing depression and 82% experience anxiety and stress [3], compared to a combined rate of approximately 17% [4] in the general population. However, patients with rare conditions are also frequently misdiagnosed as having psychosomatic illnesses [1] when their symptoms are difficult to align with a common condition. As such, the relationship between rare diseases and mental illnesses is complex, and the concurrent management of the two fraught with difficulty for both doctor and patient. In this essay I shall describe the problems faced when rare diseases are mistaken for psychological conditions and how this relates to the challenges that having a rare disease poses to the maintenance of good mental health.

A major barrier to a rare disease patient receiving appropriate treatment is the delayed receipt of a diagnosis. My own condition took 17 years to be confirmed, and this is not particularly unusual. An average patient waits for four years and sees five different doctors [1], but some may wait decades and others will never receive a diagnosis. This delay prevents patients from accessing treatment and will often cause unnecessary deterioration of their condition. One reason for such a delay is that the patient is not recognised by primary healthcare professionals as having a serious physical health problem. It is common for rare disease patients to have unusual constellations of symptoms, meaning physicians do not recognise the underlying pathology. As such, patients are frequently labelled as hypochondriacs, or as having a psychosomatic disorder. For many, a note such as this on a medical file means their complaints will never be investigated seriously again. This problem with misdiagnosis is further exacerbated by the fact that many rare disease patients may present simultaneously with symptoms which are psychiatric in nature, leading the doctor to mistakenly attribute all symptoms to the genuine mental health issue. These interactions with healthcare professionals are often extremely upsetting to the patient, leaving them feeling invalidated, mistrusted, and hopeless. As such, consultations like these are a significant source of the stress a rare disease patient can experience whilst obtaining a diagnosis, and so can themselves contribute to the development of mental health problems.

Beyond these interactions with medical professionals, the life of a patient with a rare disease can often be stressful and unpredictable. This creates an ideal environment for the development of a mental health problem such as depression or an anxiety disorder [2]. Most rare disease patients regularly attend multiple clinics, often at different hospitals and on different days, sometimes many miles from their home [1]. For example, I regularly attend four clinics, and have contact with three more, sometimes travelling for over three hours to do so. These visits require me to frequently take large amounts of time out of my daily life; I am lucky in that, as a student, my schedule has some flexibility. For children at school and adults with jobs, this situation is much more difficult. Missing large portions of school can increase social isolation in children and may contribute to academic difficulties, whilst working adults may experience job insecurity and loss of earnings. All these factors increase stress and decrease quality of life.

Furthermore, the time spent on medical appointments is not just restricted to their attendance. Co-ordination of care between the several clinics any one individual attends is usually managed by the patient themselves [1], a role which can demand massive input of time and energy. This management of care also extends to the often relentless fight for funding, whether this be for the off-label use of drugs which may prove beneficial to the patient, or for personal care and mobility. The emotional demands of the processes involved in obtaining funding cannot be underestimated; the constant requests for correspondence and evidence are exhausting. However, even when all requirements are fulfilled, the result is often still disappointing, creating a cycle of hopelessness and futility.

A rare disease can also cause difficulties for a patient in many aspects of their life entirely unrelated to their medical care. Social isolation is common, as the symptoms of rare diseases can prevent patients from leaving the home freely, whilst mobility issues and practical difficulties can make certain activities inaccessible. This can be compounded by the fact that an individual’s condition is unlikely to be fully understood by those around them, and so family and friends may be inadvertently insensitive to the extent of the effects of the rare disease on the individual’s life. The delay in diagnosis can also mean that families have witnessed the patient’s symptoms for many years with no medical explanation, and so in some cases they have become accustomed to thinking the patient is “not really ill”. This invalidation can cause further distress for the patient, potentiating the other impacts of their condition.

A patient’s intrinsic sense of identity may also be significantly affected by their rare disease. They will often have no definitive prognosis, which can contribute to prolonged uncertainty and anxiety; 91% of rare disease patients reported being worried about the future outlook of their disease [3]. This makes future planning and maintaining hope difficult. Furthermore, particularly where a condition develops after early childhood, an individual with a rare disease can have to adjust their perception and expectations of themselves significantly. In my experience, this point came when I began to use a wheelchair. Whilst my doctors were were seeing X-rays, nuclear bone scans, and muscle wastage, I was seeing hobbies I could no longer participate in, exams that would be difficult to sit, and a pile of laundry that had just become exponentially more challenging. I had to learn to rely on other people in a way that was uncomfortable and unwelcome, and re-shape my perception of myself. For me, this has been the most challenging aspect of my condition to this day.

It is easier now to look at my own experiences and focus on the good; how much of a relief it was to get a diagnosis, how helpful my complicated care is, how well I have adapted to the restrictions of my condition. But my outcomes have made me one of the lucky ones, and to focus on only the good is to miss the opportunity to improve care for those who will experience a rare disease in the future. When you consider these different ways in which a rare disease can impact a patient’s life, and the self-perpetuating cycle of misdiagnosis and psychological distress, it is not difficult to see why such patients are affected disproportionately by mental illness. However, it is harder to understand why this relationship is so neglected. Only one in seven rare disease patients say they receive sufficient psychological support [1], and if we are to truly care for these patients, the availability of psychological support must be a basic treatment requirement. Perhaps more importantly, this support must be offered in a way which doesn’t make patients feel like they are being told that their illness is psychological in origin. Whilst rare diseases are so frequently mistaken for psychosomatic disorders, patients will be reluctant to do anything which may perpetuate that view, including accepting psychological help. Until this common misdiagnosis is addressed, “It’s not all in my head!” will remain the common response to offers of psychological help, and so patients will remain unsupported.
